# Tissue Engineering Strategies in Ligament Regeneration

**DOI:** 10.1155/2012/374676

**Published:** 2011-12-25

**Authors:** Caglar Yilgor, Pinar Yilgor Huri, Gazi Huri

**Affiliations:** ^1^Department of Orthopedics and Traumatology, Hacettepe University Faculty of Medicine, Sihhiye, 06100 Ankara, Turkey; ^2^Department of Biochemistry, Cukurova University Faculty of Medicine, Balcali, 01330 Adana, Turkey; ^3^Department of Orthopedics and Traumatology, Cukurova University Faculty of Medicine, Balcali, 01330 Adana, Turkey

## Abstract

Ligaments are dense fibrous connective tissues that connect bones to other bones and their injuries are frequently encountered in the clinic. The current clinical approaches in ligament repair and regeneration are limited to autografts, as the gold standard, and allografts. Both of these techniques have their own drawbacks that limit the success in clinical setting; therefore, new strategies are being developed in order to be able to solve the current problems of ligament grafting. Tissue engineering is a novel promising technique that aims to solve these problems, by producing viable artificial ligament substitutes in the laboratory conditions with the potential of transplantation to the patients with a high success rate. Direct cell and/or growth factor injection to the defect site is another current approach aiming to enhance the repair process of the native tissue. This review summarizes the current approaches in ligament tissue engineering strategies including the use of scaffolds, their modification techniques, as well as the use of bioreactors to achieve enhanced regeneration rates, while also discussing the advances in growth factor and cell therapy applications towards obtaining enhanced ligament regeneration.

## 1. Introduction

Fibrous connective tissue bands connecting two or more bones are called ligaments. Ligaments augment joint stability and resist to forces to prevent excessive motion. Extracellular matrix (ECM) forms 80% of the tissue volume and fibroblasts make up the remaining 20%. The dry weight of a ligament consists of collagen (75%), elastin (1%), proteoglycans, and glycoproteins [1]. 90% of the collagen is type I and 10% is type III.

Although ligaments sustain excessive mechanical loads, they have a poor regeneration capacity with their low cell density and low nutrient and oxygen requirements. Thus, ligaments are repaired by a weaker and disorganized tissue which is prone to reinjury [2]. Using autografts for ligament reconstruction remains the gold standard with their high mechanical strength and compatibility, besides having high revascularization and remodeling capacities [3]. However, donor site morbidity and damage, thus, pain, and altered harvest site biomechanics that sometimes require a second invasive procedure are the drawbacks of autografts [4–6]. Allografts, on the other hand, exclude the risks associated with autografts, such as donor site morbidity; however, they carry additional risks of disease transmission, infection, and allergic reactions in addition to their lower early cellularity and less revascularization [7]. These circumstances drive attention to other techniques for ligament reconstruction, such as the use of biomaterials, cell therapies, and tissue engineering strategies, to promote a more functional healing. Preserving the native insertions and proprioceptive functions of the ligaments are advantages of these techniques leading to functional healing, over the surgical reconstruction of the tissue.

Tissue engineering strategy involves the use of biodegradable and biocompatible biomaterials with adequate structural and mechanical properties to mimic the organization of the native tissue, along with cells isolated from the healthy proportion of the patients own ligament, or other alternative cell sources such as stem cells, and growth factors to regulate the function of these cells. Conceptually, tissue engineering aims to improve the quality of the processes associated with the healing of the ligaments by creating viable artificial substitutes in the laboratory and their transplantation to the patient after in vitro maturation. Therefore, tissue engineering holds promise for the future in terms of decreasing the need for ligament grafting procedures while reducing the risks associated with them, such as rejection and tissue mismatch, as the construct would carry the patient's own cells.

From the clinical point of view, the main advantages offered by the use of tissue engineered ligament could listed to be minimal patient morbidity, simpler surgical technique, reliable fixation methods, rapid return to preinjury functions, minimal risk for infection or disease transmission, biodegradation at a rate that provides adequate mechanical stability, and supporting host tissue ingrowth [8]. Cellular adherence and matrix formation are also included in the design factors of ligament tissue engineering [9].

Another important aspect that should be taken into account in the clinical translation of tissue engineered ligament is the ligament-bone interface, which consists of a multilayered transition zone. The tissues involved in this interface display distinct mechanical properties; the ligament is strong in tension and bone is strong in compression [10, 11]. Therefore, interface is challenging for tissue engineers to mimic creating one of the current field of interests in ligament tissue engineering.

Anterior cruciate ligament (ACL) and medial collateral ligament (MCL) as well as the glenohumeral ligaments are the most frequently practiced ligament tissues to date, while all ligaments are in the pursuit of tissue engineering and studies are being carried out to create functional biological replacements of these tissues.

## 2. Growth Factor and Cell Therapies in Ligament Repair

Growth factors are regulators of cellular activities and several of them, including insulin like growth factor I (IGF-I), transforming growth factor-*β* (TGF-*β*), vascular endothelial growth factor (VEGF), basic fibroblast growth factor (bFGF), epidermal growth factor (EGF), and platelet derived growth factor (PDGF), were shown to be effective in the healing of ligament repair. In vitro and in vivo studies have shown that these growth factors have the capability to improve ligament cell proliferation and matrix formation alone or in combination [12–21]. Deie et al. have demonstrated that rabbit MCL fibroblasts are responsive to TGF-*β*1 and EGF [12] and Hildebrand et al. suggested that use of PDGF may improve the quality of ligament healing [13]. In a similar study, TGF-*β*1 and IGF were shown to modify the metabolic activity of cells of healing ligaments in rabbit MCL [14].Marui et al. have reported that topical application of TGF-*β*1, alone or in combination with EGF, may strengthen the ligament by increasing matrix synthesis during its healing processes [15]. Age and fibroblast origin were also found to be important factors in determining the proliferative response to PDGF and bFBF [12, 16]. Schmidt et al. also reported that PDGF, bFGF, and IGF-I can stimulate cell proliferation in ligaments [18]. Kobayashi et al. demonstrated that the application of bFGF enhances neovascularization and the formation of granulation tissue in lacerated canine ACL [22]. Kobayashi et al., in a rabbit study, have developed a quantitative method to assess cell migration and their findings supported previous qualitative observations [23]. They used a mathematical method to analyze cell densities in a wound model and showed that cells moved into cell-free areas [23]. DesRosiers et al. have combined EGF, PDGF, TGF-*β*1, and IGF-I two by two and analyzed their effects on cell proliferation and proteoglycan synthesis. Their results showed that EGF and PDGF had a greater effect than TGF-*β*1 and IGF-I on cell proliferation and proteoglycan production was increased by all four factors, with TGF-*β*1 having the strongest effect [19]. Others have demonstrated that combination of growth factors can have synergistic effects [20, 21]. Letson and Dahners demonstrated that ligaments treated with a combination of PDGF plus IGF-I and PDGF plus bFGF had increased rupture force, stiffness, and breaking energy [20]. A similar study showed that synergistic effect of combination of bFGF, TGF-*β*1, bovine insulin, and PDGF was as much as 20-fold of the effects of individual factors [21].

Platelet rich plasma (PRP) and collagen-PRP-complex (CPC) were also shown to be effective in the improvement of ligament healing. In a study of Liu et al., platelet concentration was shown to have a dose-response relationship with proliferation of mesenchymal stem cells (MSCs), fibroblasts, and production of collagen type I in vitro [24]. Thus, PRP is attributed to be an effective agent for ligament healing [25, 26]. Studies have supported this idea, showing effects of PRP on intraarticular ligament homogeneity [27], increase in load at yield, maximum load, and linear stiffness [28]. However, other studies concluded that application of PRP did not yield any advantage over standard ACL reconstruction procedures [29, 30]. In contrast, the use of CPC on ACL allografts was reported to inversely correlate with sagittal plane laxity [31]. Murray et al. used CPC to fill the ACL wound site at the time of suture repair in pigs and concluded that CPC scaffolds result in improved biomechanical properties [28].

Cell sources used in the repair of ligaments include MSCs which have revealed great potential in tissue engineering as a cell source that can differentiate into various connective tissue cell types including fibroblasts [32–35]. It was shown that MSC concentration at the site of injury can be augmented by allogenic MSCs delivered via the bloodstream [33]. Dermal fibroblasts are another possible cell source [36] which is easier to harvest and also display low donor site morbidity. Presence of such cells was reported to enhance peak breaking stress of hybrid collagen biomaterials in an in vitro study [37].

Functioning of certain cells can be altered via introducing DNA fragments using retroviral, adenoviral or liposomal carriers [38]. After successfully introducing a marker gene and detecting its expression in rabbit MCL and ACL [39], potential therapeutic genes such as TGF-*β*1, IGF-1, PDGF, and bone morphogenetic protein- (BMP-) 12 were also successfully introduced and expressed [40–43]. Steinert et al. have investigated the transfer of IGF-I genes using an adenovirus vector to a collagen hydrogel inserted between the cut ends of the ACL and reported promising results [40]. PDGF gene introduction was shown to enhance and accelerate matrix synthesis in a rat study and therefore claimed to be a useful tool for improving ligament repair [41]. Collagen hydrogels were used to augment ACL healing in a bovine model and increased cell accumulation was reported with TGF-*β*1-transferred hydrogels [42]. BMP-12 gene transfer was reported to result in a twofold increase of tensile strength and stiffness of repaired tendons in a chicken model [43].

## 3. Scaffolds in Ligament Reconstruction

Scaffolds are important components of tissue engineering strategy as they define the ultimate shape of the construct while providing the required mechanical strength during regeneration and proper cell attachment sites. Although there are alternate views on the ideal material, and the structure and composition of it, for ligament tissue engineering, it is generally believed that a scaffold that allows immediate load bearing and degrades at a comparable rate with the tissue regeneration would form the ideal engineered ligament [9].

Natural and synthetic materials have widely been used as ligament replacements in the forms of gels, membranes, or 3D scaffolds. Collagen, silk, hyaluronic acid (HA), ECM bioscaffolds such as porcine small intestine submucosa (SIS), and urinary bladder membrane (UBM) and polyhydoxyalkanoates (PHAs) such as poly(*β*-hydroxybutyrate) (PHB), poly-3-hydroxybutyrate-co-hydrovalerate (PHBV), and poly-3-hydroxy-10-undecenoate (PHUE-O3) are examples of potential natural replacements, whereas Dacron polyester, poly(glycolic acid) (PGA), poly(L-lactic acid) (PLLA), poly(lactic acid-co-glycolide) (PLGA), high molecular weight PLGA (HMW PLGA), poly(*ε*-caprolactone) (PCL), poly(ethylene oxide) (PEO), and poly(urethane urea) (PUUR) are examples of synthetic materials.

Collagen was one of the first natural scaffold materials to be used in ligament reconstruction as it is the natural component of the native tissue and has great ability to support ligament fibroblast growth under static tension [44]. However, collagen scaffold alone was found to be ineffective to enhance suture repair of the ACL [45]. Fibroblast-seeded collagen scaffolds, on the other hand, were more effective in ligament regeneration [44, 46–48]. It was shown in vitro that such scaffolds are consolidated with ECM and that DNA content increased rapidly over the first weeks [46]. In vivo studies have shown that fibroblast-seeded ligament analogs remain viable after implantation into the knee joint [47, 48]. Collagen scaffolds were also combined with PRP [25, 28] and various autologous and allogenic cell types [49, 50] to achieve enhanced mechanical properties and repair.

Silk was also effectively used as a ligament replacement material. It has a relatively slow rate of degradation within the body compared to collagen and other most widely used natural biomaterials which could possess an advantage in load-bearing applications. Its main advantage is its remarkable tensile strength and toughness compared to most natural materials although being lower than native human ACL [51]. The use of pure silk was shown to include problems associated with the sericin protein it contains as it may lead to allergic reactions [52]. This issue was tried to be overcome by the use of virgin silk, in which this allergen was extracted [53, 54].

Silk fibroin is a protein excreted by silkworms and isolated from sericin [55]. This protein has surface amino acids for cell adhesion and slowly degrades in aqueous solutions [56]. It can be fabricated into gels, films, and fibers. In animal models, silk fibroin has been reported to regenerate ligaments, thus claimed to be an excellent natural biomaterial alternative to collagen [9]. MSC-seeded silk fibroin scaffolds [57] and hybrid silk fibroin-silk sponge scaffolds [58] were also developed and demonstrated to be good alternatives for in vivo ligament replacement.

Composite natural scaffolds have also been fabricated using silk and collagen and then seeded with cells [59]. Due to its rapid tissue ingrowth, this chimeric silk-collagen sponge matrix was suggested to be an effective treatment for MCL transactions [59].

ECM bioscaffolds such as SIS and UBM are composed of collagen and contain cytokines and growth factors [60, 61]. ECM bioscaffolds were found to support ligament regeneration and repair and claimed to be effective candidate tools for ligament tissue engineering [62–67]. Dejardin et al. used SIS to promote regeneration of large fascial defects in adult dogs and reported promising results [62]. In a goat model, Badylak et al. reported that SIS holds promise as a resorbable bioscaffold for ACL repair [63]. MCL was shown to have better mechanical properties when SIS is applied in the healing process [64, 65]. Musahl et al. reported that SIS treatment improves not only the mechanical properties but also the histological appearance of the MCL [66]. In a more recent study, Woo et al. demonstrated that SIS enhances the fibril morphology and the collagen composition of healing MCL in rabbits [67].

In a rabbit study, Wiig et al. bilaterally lacerated ACL in the midsubstance and injected hyaluronic acid in one and saline to the other knee. The results showed increased synthesis of type III collagen in the hyaluronic acid treated injured ACL [68]. Recently, it was reported that chitosan-hyaluronan hybrid fibrous scaffolds enhance type I collagen production and improve mechanical strength in the engineered ligament in vivo [69]. PHB, PHBV, and PHUE-O3 were also reported to be good candidates for ligament tissue engineering [70].

The use of synthetic polymeric biomaterials has several advantages over the natural ones, such as they are more readily available, can be produced in large scale with low cost, and are easier to process. Moreover, their mechanical strength is mostly higher compared to natural biomaterials which offer an advantage in the engineering of tissues which are required to handle mechanical forces such as ligaments. On the other hand, they may have some disadvantages such as involving unnatural degradation byproducts and may lack functional chemical cellular binding groups [9].

Among the synthetic polymeric biomaterials used in ligament regeneration, Dacron, which is basically poly(ethylene terephthalate) (PET), is a nondegradable ligament prosthesis. It was shown that seeding of fibroblasts allowed for a more uniform connective tissue encapsulation [71].

Polyhydroxyesters that degrade by hydrolysis such as PLLA and PGA are biodegradable polymers that are popularly used in ligament repair [72, 73]. Braided PLGA scaffolds were claimed to have great promise for ligament engineering [74] while PLLA scaffolds were shown to be a more appropriate choice for ligament tissue engineering because of their slower degradation rate [75]. HMW PLGA was reported to allow more MSC attachment and proliferation than PLGA [76]. To optimize degradation rates and hydrophilicity that determines cell adhesion, composites of these materials are often fabricated [77].

A newer synthetic polymer, PUUR, was shown to have a similar loading profile as a postmortem-tested human ACL [78], and no relaxation or fatigue was observed after 50 repetitive cyclic loading [79]. PUUR was reported to keep at least 50% of its original tensile strength at body temperature for more than 9 months. Taking into account together with the strength and stiffness properties, PUUR was claimed to fulfill the desired properties for ACL reconstruction [80]. It was also shown that native cells have migrated into the implanted PUUR and that neovascularization between its fibers was detected, indicating that it is well tolerated by the host [79]. In a rabbit study where PUUR was used as a full ACL prosthesis, the knee function was reported to be intact even after 24 months [79].

## 4. Engineering the Ligament-Bone Interface

The ligament-bone interface consists of four distinct but continuous regions: ligament, noncalcified fibrocartilage, calcified fibrocartilage, and bone [81–83]. It is well known that the native interface is not regenerated in case of an injury [84, 85]. For recreating this multi-zone organization, it is essential to have a stratified or multi-phased scaffold that exhibits a continuing increase in mechanical properties through the scaffold phases [86]. In addition to such stratified, multiphased or 3D braided scaffolds, stem cell applications, cytokines, BMP-2, and BMP-12 are also considered in order to improve regeneration of this interface.

Coating of tendons with calcium phosphate layer [87], TGF-*β* [88], and BMP-2 [89–91] was found to improve osteointegration between ACL and bone tunnel, however not the fibrocartilage interface.

Multiphased, porous knitted silk [92], 3D braided PLLA [93], and poly(ethylene glycol) hydrogel incorporating HA scaffolds [94] were engineered to mimic ligament-bone interface and promising results were reported. Another innovative triphasic scaffold that has three distinct yet continuous phases including chondrocytes along with fibroblasts and osteoblasts was developed intending to regenerate the fibrocartilagineous interface [95, 96]. A further engineered ligament equivalent is reported to be a fibroblast-embedded fibrin gel with cast brushite anchors [97]. A multiphase tissue-engineered construct for ACL grafts using bone marrow origin MSCs was presented as a viable option for ACL replacement [98] using sheep as a model system.

## 5. Physical and Chemical Modification of Ligament Tissue Engineering Scaffolds

It is important to optimize cell-biomaterial interactions to achieve enhanced regeneration, mainly in terms of cell attachment and ability of cell proliferation and matrix secretion. Cell surface integrin receptors typically mediate cell-matrix interactions and the most common peptide sequence is arginine-glycine-aspartic acid (RGD) which has been used in a number of studies to achieve enhanced cell attachment [99].

In general, phosphate, amide, and sulfonate groups are used as functionalizing groups in tissue engineering applications [9]. To enhance scaffold-mediated tissue repair, growth factors are also used for signaling [100]. In a porcine model, it was shown that adding CPC to a suture repair enhances biomechanical and histological properties of the ACL via increasing cellularity within the healing ligament [26]. Silk fibroin was functionalized with MSC seeding and blending it with hyaluronan [101]. Functionalization of PET scaffolds was done with poly(sodium styrene sulfonate) (PNaSS) which was reported to have more fibroblastic adherence than nonfunctionalized fibers [102].

## 6. Bioreactors in Ligament Tissue Engineering

The body itself can be used as a “bioreactor” when cell-scaffold composites are directly implanted into the injured site or ex vivo bioreactors can be used for a period of time to achieve mature constructs prior to transplantation [103]. Ex vivo bioreactors allow application of controlled biochemical and physical regulatory signals to guide differentiation, proliferation, and tissue development [103].

Initial bioreactors applied uniaxial forces to tethered constructs [104]. More recent bioreactors are capable of applying multiaxial and cyclic strains, which better mimics the native physiology [8, 105–107]. Although the use of such bioreactor systems is a relatively new practice in ligament tissue engineering, the results are promising, which positively affects the cell proliferation and differentiation of stem cells towards musculoskeletal lineages in most if not all cases [9]. However, type, magnitude, and duration of mechanical stimuli and, thus, the ideal stimulation regime have not yet been described [9].

Application of mechanical loading was reported to have positive effects on cellular proliferation in various studies [104, 108, 109]. Mechanical loading was also shown to effect cellular morphology and alignment [104, 108–111]. The differentiation of MSCs in the presence of mechanical loads was shown to tend towards the ligament lineage [108, 112–115]. ECM synthesis and remodeling [109, 116–120] is another factor that is shown to be affected by mechanical loading. Studies have also reported that enzyme activity and growth factor expression [109, 121–123] and (6) Collagen type I, collagen type III, elastin, and tenascin-C expression in MSCs [108, 112–114] were increased with the application of mechanical loads.

## 7. Conclusion and Future Directions

This manuscript provides an overview of the previous applications and current concepts in ligament tissue engineering. Combining different approaches seems to be mandatory in order to assemble ligament-like tissue structures. Such combinations may include braiding, stratifying, knitting, or 3D braiding scaffolds as well as merging scaffolds with sponges, merging different material types in a single multiphased scaffold, aligning or cross-linking its cellular content, functionalizing its surface, and adding mechanical ex vivo stimulation. Scaffolds further need to possess a porous structure and full pore interconnectivity to allow ingrowth of native cells and connective collagen fibers.

Biomimicry is the main strategy with the intention of developing functional artificial tissues in tissue engineering. To mimic the structure as well as function of the native tissue, the interface is a great challenge and multiphased scaffolds constitute a promising option to fulfill this need.

Various types of growth factors, stem cells, cytokines and plasma ingredients; gene delivery; a range of natural and synthetic materials, and effects of mechanical loading and functionalizing have been and are being investigated. Clearly, much work remains but there are exciting and promising advances. Important future targets should include developing scaffolds that match tissue ingrowth rate with its degradation rate, matching native biomechanical properties, and should have improved strength and biological integrity as well as being able to mimic the properties of tissue interfaces.

Although many steps have been taken, to date, tissue engineering is probably still far from producing the ideal bioscaffold to replace, repair, or regenerate injured ligaments. Clinician-scientist coordination is indispensable for achieving such a goal. Along with this multidisciplinary approach, interdisciplinary contribution from biologists, chemists, biomaterial scientists, and tissue engineers is needed for meeting patients' demands.
